# Recent Research and Developing Trends of Wearable Sensors for Detecting Blood Pressure

**DOI:** 10.3390/s18092772

**Published:** 2018-08-23

**Authors:** Toshiya Arakawa

**Affiliations:** Department of Mechanical Systems Engineering, Aichi University of Technology, Gamagori, Aichi 443-0047, Japan; arakawa-toshiya@aut.ac.jp; Tel.: +81-533-68-1135

**Keywords:** blood pressure, IEEE1708, cuffless, wearable, health

## Abstract

Blood pressure is considered an index to measure a person’s health or state. The IEEE published a standard for wearable cuffless blood pressure measuring devices, which was certified as IEEE1708 on 26 August 2014, and, according to this standard, the development of wearable devices based on blood pressure is expected in the future. Considering this, blood pressure should be detectable all the time and everywhere, and this can help improve health consciousness. In this review, we introduce the recent development of wearable blood pressure measuring devices and research trends, and present the future prospects for blood pressure measuring devices.

## 1. Introduction

There is a growing awareness of the importance of lifestyle in achieving a healthy status [1]. As such awareness increases, the basic lifestyles of all people are changing. This is related to national strategies and, thus, the possibility of new business developments related to healthcare has attracted significant attention. In particular, high blood pressure can cause major diseases and ailments such as strokes and heart and kidney diseases [2,3]. Considering the rapid progression of the aging population and a Westernized diet [4,5], it is becoming increasingly important to prevent the occurrence of hypertension.

A study based on analysis data from the 2011 to 2014 National Health and Nutrition Examination Survey (n=9623) shows that, according to the criteria from the 2017 American College of Cardiology/American Heart Association (ACC/AHA) and the Seventh Report of the Joint National Committee on Prevention, Detection, Evaluation, and Treatment of High Blood Pressure (JNC7) guidelines, the crude prevalence of hypertension among US adults was 45.6% (95% confidence interval (CI): 43.6–47.6%) and 31.9% (95% CI: 30.1–33.7%), respectively. In addition, antihypertensive medication was recommended for 36.2% (95% CI: 34.2–38.2%) and 34.3% (95% CI: 32.5–36.2 %) of US adults, respectively [6]. Another report shows that one in three adults in the United States has high blood pressure, and half of them do not have it under control [7].

Another study of the relation between hypertension and economic loss showed that the total cost of treating hypertension in the United States in 203 will be US$50.3 billion—US$37.2 billion in direct medical costs, and US$13.1 billion in indirect costs owing to lost productivity related to morbidity and mortality [8]. Based on this study, the total direct cost of hypertension is followed by coronary heart disease (CHD; US$61.2 billion) and the total indirect costs of lost productivity is followed by CHD (US$129.9 billion) and stroke (US$51.2 billion). Thus, it is found that hypertension leads to decreased productivity and economic loss.

Hypertension brings not only decreased productivity and economic loss but is also a major cause of traffic accidents. There are many professional drivers whose previous symptoms, including hypertension or arteriosclerosis, worsen owing to an irregular working rotation or short break times, and there have been many cases of incapacitation and/or death from overwork [9]. It is therefore necessary for professional drivers to manage their own health condition each day, and to become aware that they should not drive without rest. In addition, it is desirable to develop a system that manages the driver’s health and prevents them from suffering undesirable conditions or sudden death, thereby decreasing the number of traffic accidents.

A previous report [7] also stated that the AHA and other organizations have called for greater use of home blood pressure monitoring; however, it is not yet widespread. One reason is that insurance coverage for such programs still lags, and another is that full-fledged efforts such as that in Minnesota could cost US$1350 per person. However, this report also states that everyone can buy a good home blood pressure monitor at a pharmacy or online merchant for anywhere from US$50 to US$100 and blood pressure should be checked twice a day for a week by the blood pressure monitor.

Considering the above, blood pressure monitors have been developed and sold widely and, in particular, non-invasive blood pressure monitors based on cuff occlusion are in wide use both inside and outside of care facilities [10]. However, these non-invasive blood pressure monitors have some disadvantages including discomfort for the patient because of painful cuff inflation, which may influence blood pressure outcome, and the unfeasibility of continuous or semicontinuous blood pressure monitoring owing to the necessity for cuff inflation and deflation.

Thus, non-invasive cuffless blood pressure measurement devices have begun to be realized and mass-produced. In fact, the standardization of a cuffless blood pressure measurement system that can measure blood pressure based on the pulse wave propagation time without a cuff has been considered, and the IEEE published a standard for wearable cuffless blood pressure measuring devices, which was certified as IEEE1708 on 26 August 2014. According to this standard, the development of wearable devices based on blood pressure is expected in the future [11].

**Figure 1:**
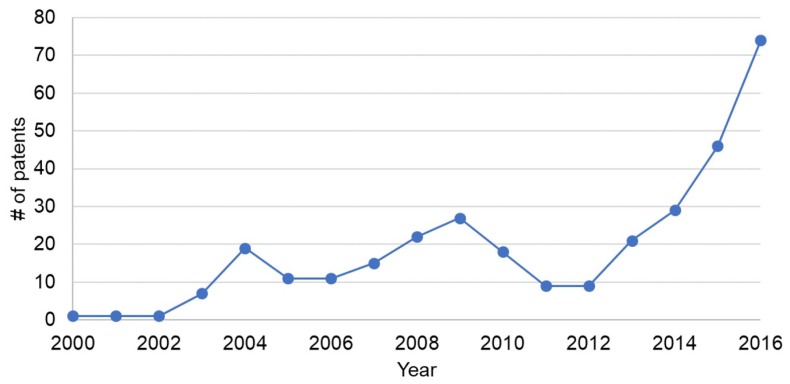
Number of patents for cuffless blood pressure monitors.

We surveyed the number of published patents registered from 2001 to 2016 on 15 August 2018. We used Google patent for our survey and we used the keywords “blood”, “pressure”, “cuffless”, and “monitor” to search for patents. As for patents in 2017, the number of published patents is still relatively small, thus we surveyed published patents from 2001 to 2017. The results are shown in Figure 1. It can be seen that the number of patents has been increasing from 2012 and there were over 70 registered in 2016. From this survey, it is found that the development of cuffless blood pressure measurement monitors has been thriving and the need for cuffless blood pressure measurement monitors has been increasing.

In this paper, the trends of non-invasive cuffless blood pressure monitors are introduced and the merit and the recent development of wearable blood pressure measuring devices and research trends are discussed. In addition, we describe the future prospects for blood pressure measuring devices. The remainder of this paper is organized as follows. Section 2 comprehensively explains the basic method for measuring blood pressure, Section 3 presents an example of a non-invasive blood pressure measuring sensor, Section 4 explains the disadvantages and problems of non-invasive cuffless blood pressure measuring systems, Section 5 describes the future prospects of blood pressure measuring systems, and Section 6 summarizes the paper.

## 2. The Traditional and Basic Methods of Measuring Blood Pressure

In this section, the traditional and basic methods of measuring blood pressure are explained. Most of the descriptions in this section are from Ogedegbe and Pickering [12].

### 2.1. Location of Measurement

The standard location for blood pressure measurement is the brachial artery. Monitors that measure pressure at the wrist and fingers have become popular, but it is important to realize that systolic and diastolic pressures vary substantially in different parts of the arterial tree with systolic pressure increasing and diastolic pressure decreasing in more distal arteries.

### 2.2. The Auscultatory Method

Although the auscultatory method using a mercury sphygmomanometer is regarded as the gold standard for office blood pressure measurement, the widespread implementation of bans in the use of mercury sphygmomanometers continues to diminish the role of this technique [13]. The situation is made worse by the fact that existing aneroid manometers, which use this technique, are less accurate and often need frequent calibration [13]. New devices, known as “hybrid” sphygmomanometers, have been developed as a replacement for mercury devices. Basically, these devices combine the features of both electronic and auscultatory devices such that the mercury column is replaced by an electronic pressure gauge, similar to oscillometric devices, but the blood pressure is taken in the same manner as a mercury or aneroid device, by an observer using a stethoscope and listening for the Korotkoff sounds [13].

### 2.3. The Oscillometric Technique

The idea behind this method is that when the oscillations of pressure in a sphygmomanometer cuff are recorded during gradual deflation, the point of maximal oscillation corresponds to the mean intra-arterial pressure [14,15,16]. The oscillations begin at approximately systolic pressure and continue below diastolic, so that systolic and diastolic pressure can only be estimated indirectly according to some empirically derived algorithm. This advantages of this method are that there is no need to place a transducer over the brachial artery, it is less susceptible to external noise (but not to low-frequency mechanical vibration), and the cuff can be removed and replaced by the patient during ambulatory monitoring, for example, allowing them to take a shower. The main disadvantage is that such recorders do not work well during physical activity when there may be considerable movement artifacts. The oscillometric technique has been used successfully in ambulatory blood pressure monitors and home monitors. It should be pointed out that different brands of oscillometric recorders use different algorithms, and there is no generic oscillometric technique. Comparisons of several different commercial models with intra-arterial and Korotkoff sound measurements, however, have shown generally good agreement [17,18].

### 2.4. Ultrasound Techniques

Devices incorporating this technique use an ultrasound transmitter and receiver placed over the brachial artery under a sphygmomanometer cuff. As the cuff is deflated, the movement of the arterial wall at systolic pressure causes a Doppler phase shift in the reflected ultrasound, and diastolic pressure is recorded as the point at which diminution of arterial motion occurs. Another variation of this method detects the onset of blood flow at systolic pressure, which has been found to be of particular value for measuring pressure in infants and children [19]. In patients with very faint Korotkoff sounds (for example, those with muscular atrophy), placing a Doppler probe over the brachial artery may help to detect the systolic pressure, and the same technique can be used for measuring the ankle–brachial index, in which the systolic pressures in the brachial artery and the posterior tibial artery are compared, to obtain an index of peripheral arterial disease.

### 2.5. The Finger Cuff Method of Penaz

Arterial pulsation in a finger is detected by a photoplethysmograph under a pressure cuff. The output of the plethysmograph is used to drive a servo-loop, which rapidly changes the cuff pressure to keep the output constant, so that the artery is held in a partially opened state. The oscillations of pressure in the cuff are measured and have been found to resemble the intra-arterial pressure wave in most subjects. This method gives an accurate estimate of the changes of systolic and diastolic pressure when compared with brachial artery pressures [20]; the cuff can be kept inflated for up to 2 h. It is now commercially available as the Finometer and Portapres recorders and has been validated in several studies against intra-arterial pressures [21,22]. The Portapres recorder enables readings to be taken over 24 h while the subjects are ambulatory, although it is somewhat cumbersome [23].

### 2.6. Need for Cuffless Blood Pressure Measuring Systems

The methods for measuring blood pressure are categorized in Table 1. Various methods of automated blood pressure measurement have been developed to decrease the load of healthcare workers. Table 1 also lists the advantages and disadvantages of each method. These methods are basically applied when users sit down on a chair with a backrest after a few minute’s calm in a quiet environment such as a hospital or at home [24]. In addition, Table 1 lists the precision of each method. Precision of catheter, Korotokoff and oscillometric is said to be at the tolerance level. On the other hand, tonometry and vascular-volume compensation is said to be at a good level [25]. Table 2 lists the difference of precision of each method.

On the other hand, blood pressure always fluctuates over the course of 24 h. Thus, hypertensive patients need to constantly monitor blood pressure to take care of their health. Ambulatory blood pressure monitoring (ABPM) devices have been developed in response to such needs and, in Japan, insurance points have been applied to these devices since 2008.

However, these devices can measure blood pressure when users wind a cuff for sphygmomanometry, and this can lead to an increase in stress for users because their movement is restricted when measuring blood pressure and they feel discomfort from the cuff. Thus, the need to develop and produce cuffless blood pressure measurement systems is clear.

Denmark-based medical device company Sense A/S (Hovedstaden, Denmark) argues that measuring blood pressure at a physicians’ office via the cuff method has a number of challenges including discomfort with the measurement itself and nervousness during the consultation, which both may contribute to erroneous measurements [26]. In addition, Sense A/S also argues that a variety of cardiovascular diseases can be diagnosed much better by measuring blood pressure over a standard 24-h circadian rhythm, and, for this, the cuff method is not very suitable [26]. In fact, in 2012, Sense A/S received a US$5.9 million round of funding from venture fund SEED Capital (Hovedstaden, Denmark) and Vaekstfonden (Hellerup, Denmark) to help it refine its cuffless blood pressure measurement device [26].

## 3. Development Cases of Cuffless Blood Pressure Measuring Systems

In this section, the development cases of cuffless blood pressure measuring systems are explained. There are mainly two types of cuffless blood pressure measuring systems: portable blood pressure measuring systems and steering-type blood pressure measuring systems. We introduce some types of systems here, but we cannot go into the details of measuring theory or the methods of all systems because they may not necessarily have been disclosed.

### 3.1. Portable Blood Pressure Measuring Systems

Here, we introduce portable blood pressure measuring systems.

#### 3.1.1. Wearable Sensor by Shuzo

This system was developed for measuring blood pressure during exercise [24]. The merit of this system is that it can be attached to portable devices, such as smartphones or portable music players, to complement their functions. Thus, this system can measure a user’s blood pressure without them being aware that they are carrying a special measurement device. In addition, users can obtain feedback and recommendations from this system such as a graphical exercise menu based on the acquired data.

The algorithm of this system is said to be based on the pulse wave velocity method. In the pulse wave velocity method, the timing of contraction of the heart and ejection of blood is considered almost the same, and the systolic blood pressure value is calculated based on the pulse wave propagation time, which is the time difference between the R wave and pulse wave rising point. Blood pressure is estimated from this result. It is shown that the pulse wave velocity, *v* (m/s), is related to the elastic modulus of the circumferential blood vessel wall, *E*, from the Moens–Korteweg equation [27]:

(1) $$v=\frac{\Delta x}{T_{PTT}}=\sqrt{\frac{Eh}{2\rho r}}.$$

Experimentally, the relation between blood pressure and vascularity is found from the following equation:

(2) $$E=E_{0}e^{\alpha P},$$

where Δx (m) denotes the measurement distance between the electrocardiogram (ECG) and pulse wave, $T_{PTT}$ (s) is the pulse wave propagation time, *h* (m) denotes the vascular wall thickness, ρ (kg/m^3^) denotes the blood density, *r* (m) denotes the blood vessel inner diameter, $E_{0}$ (mmHg) denotes the elastic modulus of the circumferential blood vessel wall without pressure, α denotes the coefficient depending on the blood vessel, and *P* (mmHg) denotes the inner pressure of the blood vessel. It is known that, from Equations (1) and (2), the relation between $T_{PTT}$ and the systolic blood pressure, $P_{S}$ [mmHg], is as follows [28]:

(3) $$P_{S}=a_{1}\ln T_{PTT}+a_{2},$$

where $a_{1}$ and $a_{2}$ are coefficients.

As to this developed system, the change of blood vessel inner diameter is focused to ensure the validity on exercise, thus the following equation of the equilibrium of forces in infinitesimal area (central angle is Δθ [rad]) on a blood vessel wall is considered:

(4) $$\Delta Pr\Delta \theta =2E\left(\frac{\Delta r}{r}\right)h\sin\frac{\Delta \theta }{2}.$$

Using Equation (4), the following Equation (5) is acquired:

(5) $$P_{S}=b_{1}\log\left(b_{2}-\frac{b_{3}}{T_{PTT}^{2}}\right).$$

This system’s target device is an iPod Touch${}^{®}$ (Apple, Inc., Cupertino, CA, USA) and an electronic circuit board and data processor are contained within the auxiliary battery, which can be attached to the iPod Touch${}^{®}$. Thus, the user can measure their blood pressure while listening to music or looking at websites. The size of this system is 65 mm width × 129 mm height × 24 mm depth and the weight of this system is 230 g (the weight of iPod Touch${}^{®}$ 112 g and Li-ion battery 18 g are included). This system can measure for 2 h continuously under a sampling frequency of 1 kHz. It can measure for 28 h if measurements are performed every 30 min under intermittent measurement.

#### 3.1.2. HeartGuide by Omron Corp.

Omron Corp. (Kyoto, Japan) publicized that it has a technique modernized to monitor blood pressure, that is, “Project Zero monitor 2.0” [29]. The wrist monitor, called “HeartGuide”, measures blood pressure with clinical accuracy at the push of a button [30]. It will measure physical activity and sleep, and sync with an Omron corp. app to track and share vital data about blood pressure. Omron Corp. has been trying to develop invasive methods for blood pressure measurement depending on the transit time of the pulse. The technology to measure blood pressure has changed over the years [29]. The watch has an extra-stiff band that actually inflates to take an oscillometric measurement in the same way as a normal doctor’s blood pressure cuff [31]. The watch takes manual readings and spot heart rate measurements but can also be programmed to take night readings to test for hypertension and the risk of stroke while sleeping [31].

This watch also tracks steps and sleep, and can also display phone notifications. This watch will last between 10 and 14 days on a single charge, according to Scott [31], which will make it easier for elderly patients to use. This watch takes manual readings and spot heart rate measurements, but can also be programmed to take night readings to test for hypertension and the risk of stroke while sleeping. It is said to take about 1 min to measure blood pressure using this watch. The height of the wrist needs to be the same as the heart to assure accuracy of measurement. The height of the wrist and heart are almost the same during sleep, thus the measurement during sleep by this watch is said to be the ideal condition [32].

We cannot strictly call this watch a cuffless device. However, the portability and the existence of the cuff is not very noticeable, thus we consider this watch almost the same as a cuffless device.

#### 3.1.3. Blood Pressure Monitor by EchoLabs

EchoLabs (Englewood, CO, USA) has invented a technology that pulses light through the tissue to the bloodstream and analyzes the reflection. The technology leverages spectroscopy to analyze blood, using transmitters to send light and other electromagnetic frequencies to the skin and measuring the light that reflects back [33]. The reflectance is not only from skin. When an electromagnetic wave is beamed into the tissue, the light is reflected from every element it hits: the arteries, skin, bone, and muscle. This monitor can read the reflectance with a receiver and can analyze how blood flow is functioning and, in addition to heart rate, this system can measure things such as blood gas concentrations [33].

This system is said to focus on accuracy. The raw signal we receive from the optical sensor is very noisy, i.e., the signal-to-noise ratio is 1 to 100. EchoLabs has explained that their system can achieve very accurate results based on the analysis of well-known metrics such as pulse transit time; however, the details of the algorithm have not been released.

### 3.2. Steering-Type Blood Pressure Measuring Systems

Thus far, we have introduced application examples of wearable blood pressure monitors. Here, we consider the situation when driving. Many traffic accidents nowadays are caused by sudden changes in physical condition and the affect they have on driving. A survey has shown that the rate of traffic accidents caused by driver sickness is from 0.3% to 3.4%. In particular, in Japan, heart disease is the cause of 23.2% of professional drivers’ sickness followed by stroke that is the cause in 28.4% of cases [34]. In addition, adequate preventative behavior was not taken in 73.5% of traffic accidents caused by professional drivers. Thus, the development of blood pressure measuring devices that can be deployed when driving is expected. One solution to this is the system explained above; however, if the driver forgets to wear the devices, blood pressure cannot be measured, thus it is desirable to measure driver’s blood pressure when driving without wearing a special device. Thus, steering systems that can measure the driver’s blood pressure when driving have been developed. In addition, a previous study has shown that a driver’s surprised state when facing an incident situation can be detected by blood pressure measurement [35]. Thus, steering-type blood pressure measuring systems have the possibility to detect both the driver’s health and their state when driving.

In this section, we introduce the steering-type blood pressure measuring device.

#### 3.2.1. Cuffless Blood Pressure Monitoring Using Steering Wheel Sensor System by Denso Corp.

Denso Corp. (Kariya, Aichi, Japan) has developed a steering wheel sensor system for cuffless blood pressure monitoring [36,37]. To monitor blood pressure while driving, an EEG (Electroencephalogram) sensor and optical sensor are installed in the steering wheel. Two pairs of chrome-coated electrodes (contact impedance is 90 kΩ and input impedance is 1000 kΩ [37]) are located on the steering wheel. Each pair of electrodes is composed of a ground electrode and a signal electrode. When the driver grips the steering wheel, the system detects voltage between both hands derived from a movement of heart muscle, or ECG. Different from medical sensors, no gel should be used. To reduce common mode noise from dry metal electrodes, input impedance of the circuit is designed to be high enough to receive an adequate signal. An optical sensor composed of a 525 nm green LED and PD similar to conventional medical sensors detects the pulse wave from under the skin of the driver’s palm. The reason why a 525 nm green LED is used is to reduce surface reflection from the skin surface and therefore enhance the signal-to-noise ratio of the pulse wave.

To estimate blood pressure precisely, the pulse wave is analyzed and cardiovascular-related features are extracted. The percussion wave, tidal wave, notch, and dicrotic wave are obtained from the pulse wave [38]. Six peak values from acceleration plethysmography are obtained by this system [39].

Considering the result of experiments using this system, the system’s accuracy satisfied the requirements of a sphygmomanometer in the under-sixties class, whereas the accuracy is poor in the over-sixties class.

#### 3.2.2. Steering-Type Blood Pressure Monitoring System by Arakawa et al.

We developed a steering-type blood pressure measuring system to measure blood pressure when driving [11,40]. The features of this system are that it is cuffless and robust to body movement on turning the steering wheel. However, we consider this development a feasibility study, and the system was created for a desktop driving simulator, not for a real vehicle. Thus, we developed the system to measure blood pressure while playing a racing game on a PlayStation 4${}^{®}$. A steering controller for a PlayStation 4${}^{®}$ (Hori Co., Ltd. (Yokohama, Kanagawa, Japan)) was used as the steering wheel. This steering-type sphygmomanometer is connected to a tablet PC through Bluetooth. The blood pressure data measured by the sphygmomanometer are transmitted to the tablet PC, which displays the driver’s blood pressure in real time.

**Figure 2:**
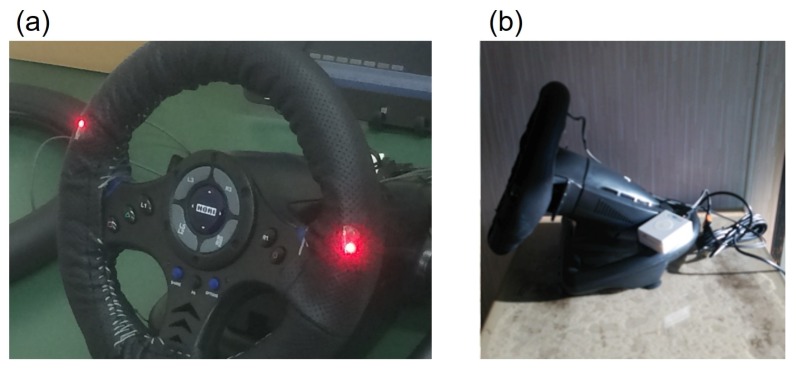
Steering-type blood pressure monitoring system by Arakawa et al.: (**a**) front view of the system and (**b**) side-on view of the system from the right.

**Figure 3:**
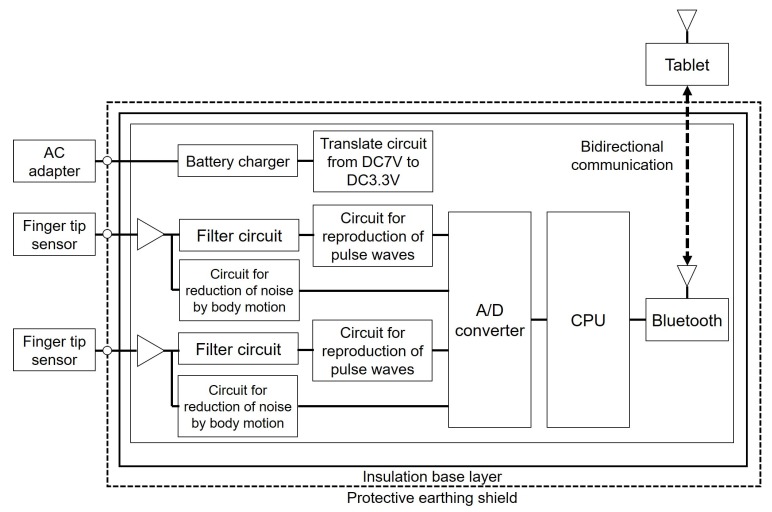
Block chart of the developed system.

Figure 2 shows the developed system and Figure 3 illustrates a block chart. Two sensors are attached to the steering wheel spokes because the steering wheel turns from $-90^{\circ }$ to $+90^{\circ }$, and the driver places their hands at almost the same location on a real steering wheel when driving. The sensors are therefore attached at positions of 10 and 2 o’clock on the wheel, and can detect the driver’s pulse even if the driver removes one hand from the wheel.

A conventional sphygmomanometer is a traditional tool for measuring blood pressure. The advantages of this device are that no transducer needs to be placed over the brachial artery, it is less susceptible to external noise (but not to low-frequency mechanical vibrations), and the cuff can be removed and replaced by the patient during ambulatory monitoring, for example, when taking a shower. The main disadvantage, however, is that such recorders do not work well during physical activity, when considerable movement artifacts may occur. An oscillometric technique has been used successfully in APBM and home monitors [12]. Thus, it is inadequate to apply a conventional sphygmomanometer for detecting the driver’s state, and application of photoplethysmography as a non-invasive sensor technique has been considered [41]. Thus, we developed a non-invasive blood pressure detection system using photoplethysmography.

With this system, infrared light is emitted from a sensor unit attached to the steering wheel ring aiming at the skin of the driver’s finger. The transition of the finger plethysmogram, which is the integral value of the photoplethysmogram, is calculated for every pulse beat, and the reference light quantity is used to determine the average blood pressure. In addition, blood flow, the condition of the hemoglobin, and the vascular elasticity rate are calculated. The systolic and diastolic blood pressures can be continuously calculated based on these results.

The algorithm for calculating the blood pressure is shown in the following [42,43]. Photoplethysmography is applied to a pipe flow in a viscous fluid (Hagen–Poiseuille flow, the algorithm of which follows the equation below):

(6) $$Q=\pi \times R^{2}\times V=\frac{\pi \times R^{4}}{8\times \mu }\times \frac{P_{1}-P_{2}}{L},$$

where *Q* (m${}^{3}$/s) is the flow volume, *R* (m) is the radius of the pipe, *V* (m/s) is the flow velocity, η (Pa·s) is the viscosity of the fluid, and $(P_{1}-P_{2})/L$ (Pa/m) is the pressure gradient between two points (*L* (m)).

In short, the pressure correlates the flow volume in a pipe. The above equation was applied for the photoplethysmography. The following assumptions were made in the logic of the estimation.

1. The blood pressure correlates positively with blood flow.
2. The mural pressure correlates positively with the pressure against a tissue (e.g., the cuff pressure or application of the probe pressure).
3. A constant probe pressure is applied to the tissue.
4. The pressure difference is higher for the arteries than for the venous vessels.
5. The photoplethysmographic signals are sensitive only to the hemoglobin dynamics.

Therefore, when adequate pressure and adequate light emission are applied to the tissue, the transmitted light is hypothesized to correlate with the blood pressure, i.e., the systolic pressure was estimated for the peak of the light transmitted and the diastolic pressure was estimated for the trough transmission of the light.

**Figure 4:**
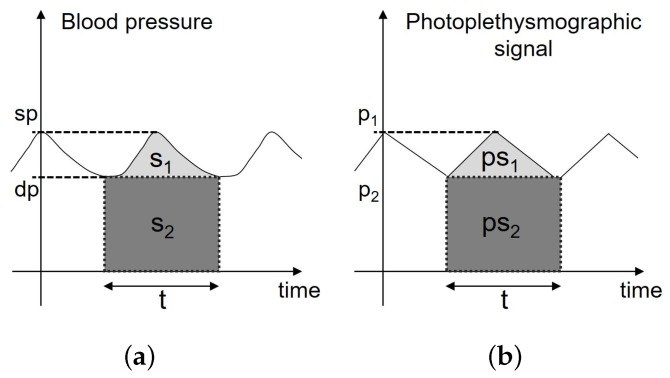
(**a**) Schema of the blood pressure pulsation and time course and (**b**) schema of the photoplethysmographic pulsation and time course.

Figure 4 shows a schema of the blood pressure pulsation and time course. The following equations were hypothesized for measuring blood pressure:

(7) $$\begin{array}{ccc} s_{1} & = & \frac{s_{p}-d_{p}}{2}\times t, \end{array}$$

(8) $$\begin{array}{ccc} s_{2} & = & d_{p}\times t, \end{array}$$

(9) $$\begin{array}{ccc} S & = & s_{1}+s_{2}, \end{array}$$

(10) $$\begin{array}{ccc} K & = & \frac{s_{1}}{s_{2}}, \end{array}$$

where $s_{p}$ (mmHg) denotes the systolic pressure, $d_{p}$ (mmHg) denotes the diastolic pressure, and *t* (s) denotes the wave period.

The following equations were then hypothesized for the photoplethysmographic pulsation:

(11) $$\begin{array}{ccc} ps_{1} & = & \frac{p_{1}-p_{2}}{2}\times t, \end{array}$$

(12) $$\begin{array}{ccc} ps_{2} & = & p_{2}\times t, \end{array}$$

(13) $$\begin{array}{ccc} pS & = & ps_{1}+ps_{2}, \end{array}$$

(14) $$\begin{array}{ccc} pK & = & \frac{ps_{1}}{ps_{2}}, \end{array}$$

where $p_{1}$ (mW) denotes the maximum photoplethysmographic signal intensity, $p_{2}$ (mW) denotes the minimum photoplethysmographic signal intensity, and *a* denotes an arbitrary constant.

The following equations below were deduced when *K* is hypothesized as pK:

(15) $$\begin{array}{ccc} ps_{1} & = & \frac{K}{1+K}\times pK, \end{array}$$

(16) $$\begin{array}{ccc} ps_{2} & = & \frac{1}{1+K}\times pK, \end{array}$$

(17) $$\begin{array}{ccc} p_{1} & = & \frac{(2K+a)\times pK}{(1+K)\times t}, \end{array}$$

(18) $$\begin{array}{ccc} p_{2} & = & \frac{pK}{(1+K)\times t}. \end{array}$$

Therefore, $p_{1}$ (mmHg) and $p_{2}$ (mmHg) are estimated as the systolic and diastolic pressures.

**Figure 5:**
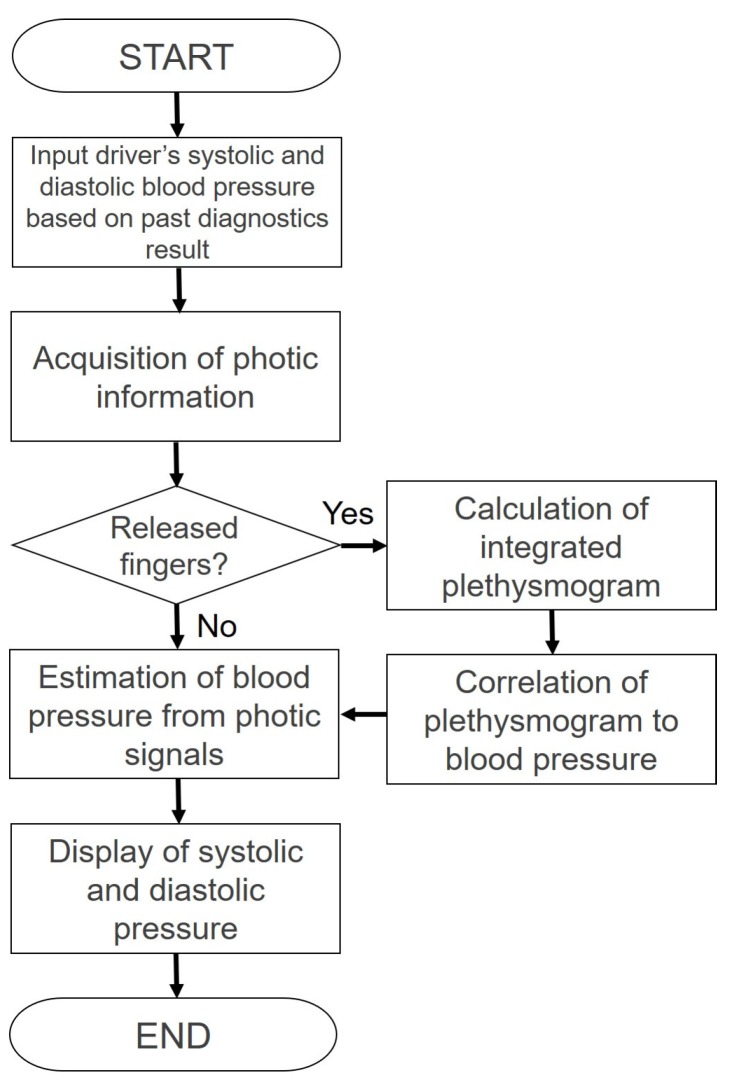
Flow chart estimating continuous blood pressure using a photoplethysmogram.

Figure 5 shows a flow chart of the blood pressure detection using the developed system. Next, we describe the calibration procedure of Figure 5.

1. As a reference to determine the upper and lower limits of their blood pressure, the drivers input their systolic and diastolic blood pressures based on past diagnostics into the tablet.
2. The pulse waves recorded when holding the steering wheel are detected to determine the reference finger plethysmogram, and the finger plethysmogram is tuned to the specified gain based on the amount of light and sensitivity.
3. The tuned photoplethysmogram is considered a standard plethysmogram with regard to the average blood pressure. The ratio of diastolic blood pressure to the average blood pressure is added to the plethysmogram of the average blood pressure, which is determined as a plethysmogram of the diastolic blood pressure. A plethysmogram of the systolic blood pressure is calculated similarly based on the ratio of systolic blood pressure to the average blood pressure, and is considered a standard plethysmogram.
4. The observed plethysmogram and plethysmogram of the standard blood pressure are compared, and the current average blood pressure is calculated. Next, the systolic and diastolic blood pressures are calculated based on the pulse pressure ratio calculated based on the ratio of height of the plethysmogram.

**Figure 6:**
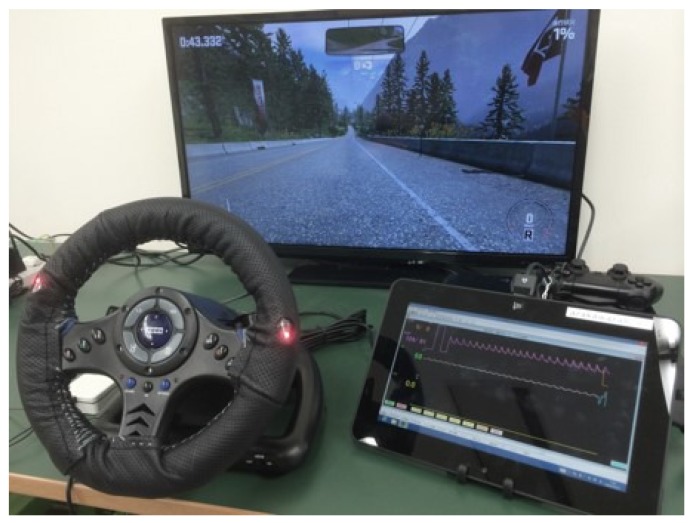
Appearance of the developed system and tablet interface.

Photoplethysmographic detection by sensors attached to the steering wheel is branched into a circuit to reduce the noise from body movements and a circuit for filtering. A photoplethysmogram passes through a circuit to reproduce a pulse wave after passing through the circuit for filtering, and the blood pressure is calculated based on a photoplethysmogram by applying digital processing. Finally, we obtain a pulse wave and the value of the blood pressure as the output. The appearance of the developed system operation is shown in Figure 6.

## 4. Comparison between Traditional Non-Invasive Blood Pressure Systems and Non-Invasive Cuffless Blood Pressure Systems

Thus far, we have introduced various developments in non-invasive cuffless blood pressure measuring systems. In this section, we describe the disadvantages and problems of non-invasive cuffless blood pressure measuring systems.

In previous sections, we have introduced the two kinds of cuffless blood pressure measuring systems: portable device types, such as watches or smartphones; and steering type for detection when driving. We now discuss the disadvantages of these system. For the portable device type, the significant disadvantage is that users always have to carry and wear them and if they forget to carry and/or wear it, they cannot measure their blood pressure. Some users might think that it is troublesome to always wear the device or put it on each time they leave the house. If users feel that it is not worth wearing them everyday, they will stop carrying or wearing them easily. One method for solving this problem is the development of underwear-like or T-shirt-like blood pressure measuring devices. For example, a T-shirt-like or vest-like pulse measuring sensor has been already developed [44,45,46]. In particular, Hitoe [47,48,49] is a T-shirt-type wearable sensor developed by NTT and Toray and can collect ECG and three-axis acceleration data of the wearer [50]. As is generally known, T-shirts are an everyday item, thus wearing a T-shirt-like device, such as Hitoe, is natural for users. Thus, if a T-shirt-like blood pressure measuring device could be developed, it would be more acceptable for users. An advantage of Hitoe is that the biometric information is transmitted to the smartphone or tablet by the transmitter attached T-shirt and can be easily confirmed with the application. New services are also expected in fields such as health promotion and sleep management, security, entertainment and others.

On the other hand, for steering-type blood pressure measuring devices, the user does not need to wear a device and their blood pressure can be measured unconsciously only when they hold the steering wheel. However, if a special circuit or hardware is not attached to the system, the system cannot measure a user’s blood pressure when the user holds the steering wheel with one hand. For example, we introduce the pulse wave measuring system by Toyota Motor Corp. (Toyota, Aichi, Japan), Denso Corp., and Nippon Medical School [51].

Severe arrhythmia and sudden myocardial ischemic attacks that occur suddenly during driving not only pose a risk of sudden death to the driver, but may also pose the risk of causing a serious car accident. Therefore, this system was developed for detecting autonomic nerve activity and changes in ECG that are considered as these signs as soon as possible. An electrode for electrocardiography measurement and an optical sensor for pulse wave measurement are mounted on the steering wheel. When the driver grasps the steering wheel, it is possible to continuously measure and record the ECG and the pulse wave. By analyzing these measurement data in real time (heart rate variability analysis), this system finds a unique pattern that appears to be a sign of sudden change in physical condition. As the electrodes for electrocardiography measurement were arranged at the positions of both the left and the right hands, the previous system could not perform measurements unless both hands were used for steering [51]. In contrast, for this system, electrodes are placed on the seating surface of the seat so that this system can measure the ECG with two electrodes, the right hand and the seating surface, even if users hold the steering with only the right hand. However, in the case of a seating surface that is different from the electrode to be placed on the steering, users touch the electrodes through clothing [51]. Thus, there is increased noise, and further revisions of system and algorithm for measuring the pulse wave will be needed. This example is for a pulse wave measurement system, not a blood pressure measurement system, but is still relevant. As described above, for steering-type blood pressure measurement system, the user’s holding steering state and noise are the major problems. In other words, it could be a useful system if these problems can be solved. The devices introduced in this paper are good examples that have solved these problems.

For both portable-type devices and steering-type devices, four factors are important for devices being introduced to market (the first three points have been addressed above): first, devices should be suited to daily use with regards to portability; second, the device should measure blood pressure in a subtle manner; third, the system should be able to perform measurements in any circumstances; and, finally, cost. The system will not be marketable or acceptable for users if the device is good but very expensive.

## 5. Future Prospects

Recently, other methods for measuring blood pressure have been presented. One interesting and notable method is measurement by ultrasonic sonar. We think this method is promising for developing a cuffless blood pressure measuring device. For example, a system has been developed that provides both control of the measurement process and analysis of the resulting flow signal [52,53]. The system is based on a PC with Matlab software (The MathWorks, Inc., Natick, MA, USA) a wristwatch ultrasound device, and a pressure cuff driven by a conventional pressure monitor. The evaluation algorithm provides the determination of systolic blood pressure, heart frequency, and signal quality in normal and common pathologic conditions (arrythmia) [54].

Comparisons with invasive measurements showed correlation (mean ΔP 3.0 mmHg, n=13) [54]. During ergometry, the difference between pressure evaluated by ultrasound and a stethoscope was reported as 4.6 mmHg (n=12).

Researchers at Tohoku University have started demonstration experiments with measuring blood pressure by ultrasonic sonar [55]. Following these experiments, Haga developed a sensor that can measure blood pressure with ultrasonic sonar [56]. The pulse wave is emitted from an ultrasonic transducer placed on the skin. Blood pressure can be measured using the reflective echo from the front and back blood vessel wall. In this system, elements that are placed over the blood vessel have been placed as a two-dimensional array to use only signal from the elements that detect changes in blood vessel diameter. This enables easy alignment of the ultrasonic transducer, which is placed on the skin, and the blood vessel, which is found under the skin. Thus, a thin and light ultrasonic transducer seat sensor can be realized [56].

**Figure 7:**
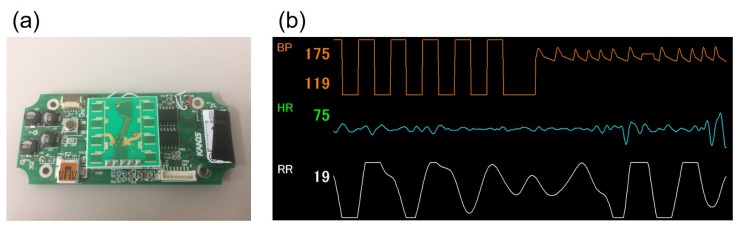
Prototype sensor that can measure blood pressure with ultrasonic sonar by Arakawa et al.: (**a**) the hardware system and (**b**) graphical user interface on measuring with this system, where the upper graph shows the time-series data of blood pressure.

The reason that these blood pressure measuring systems using ultrasonic sonar are interesting and notable is that they have freedom of design because ultrasonic sonar can even measure blood pressure from a small, distant target. Thus, for in-vehicle applications in particular, the system will not be limited to being located at the rim of steering wheel, and instead may be located at the center of steering wheel, meter visor, seat back, and so on. For example, we developed a small blood pressure measuring system using ultrasonic sonar, as an application of the steering-type system mentioned in Section 3.2.2 [57] (Figure 7). This system is intended to be located on on the meter visor or at the center of the steering wheel.

However, there are some difficulties in measuring blood pressure using ultrasonic sonar. For example, the arms of the driver may inhibit the ultrasonic sonar located at the center of steering wheel when the wheel is turned. As another example, the buffer material inside the seat may also inhibit the ultrasonic sonar when the system is located in the seat back. In fact, our pre-examination has shown that the driver’s blood pressure signal detected with this system, which was attached to the seat back, was relatively noisy because the buffer material inside the seat seemed to inhibit the signal path. Thus, there seems to be a need to develop a noise-reducing technology or blood pressure technology that is stable under all circumstances to apply these blood pressure measuring systems using ultrasonic sonar.

As for the method for calculating blood pressure and its accuracy, various studies have appeared [58,59]. Considering these previous studies, it is one of the future missions that portable devices, which everybody can use everyday and everywhere, will be developed based on the method like these previous studies.

## 6. Conclusions

In this paper, the trends of non-invasive cuffless blood pressure measurement have been introduced and the merits, recent developments, and research trends of wearable blood pressure measuring devices have been introduced. In addition, we have described future prospects for blood pressure measuring devices. There are some problems to be solved before launching to market; however, blood pressure measuring devices will be key to monitoring future health problems. We hope that this manuscript will be useful for the future development of blood pressure measurement systems.

## Figures and Tables

**Table 1:** Comparison of the methods of blood pressure measurement [24,25].

Method	Advantages	Disadvantages	Precision
Catheter	True value, continuous	Invasive	middle
Korotokoff	Non-invasive	Cuff pressure, sensitive to sound	middle
Oscillometric	Non-invasive	Cuff pressure, sensitive to movement	middle
Tonometry	Non-invasive	Cuff pressure, sensitive to movement	good
Vascular-volume compensation	Non-invasive, continuous	Cuff pressure control	good

**Table 2:** Main error factor of the methods of blood pressure measurement [25].

Cause	Error Factor	Systolic Blood Pressure	Diastolic Blood Pressure
Measurement position	Higher than right atrium	Decrease	Decrease
	Lower than right atrium	Increase	Increase
	Narrow width	Increase	Increase
Measurement with cuff	Wide width	Decrease	Decrease
	Wrap loose	Increase	Increase
Measurement method	Slow reducing pressure	Decrease	Increase
	Low hearing ability	Decrease	Increase
